# Editorial: Theory of mind in robots and intelligent systems

**DOI:** 10.3389/frobt.2025.1750134

**Published:** 2025-12-11

**Authors:** Nikolos Gurney, Dana Hughes, David V. Pynadath, Ning Wang

**Affiliations:** 1 Institute for Creative Technologies, Thomas Lord Department of Computer Science, Viterbi School of Engineering, University of Southern California, Playa Vista, CA, United States; 2 Advanced Agent - Robotics Technology Lab, Robotics Institute, School of Computer Science, Carnegie Mellon University, Pittsburgh, PA, United States; 3 The Ken Kennedy Institute, Rice University, Houston, TX, United States

**Keywords:** theory of mind, social cognition, artificial social intelligence, human robot interaction, decision theoretic models of social reasoning, bayesian theory of mind, cognitive architectures, computational models of cognition

## 1 Introduction

The hope and, in some cases, fear that intelligent machines will understand the mental states of their human counterparts, that is, have a theory of mind (ToM), has been with us since the advent of the idea that machines may 1 day be as intelligent as us. Early evidence of this was found in responses to ELIZA, Joseph Weizenbaum’s script-based agent for studying natural language communication between man and machine (Weizenbaum, 1966). Weizenbaum’s study participants reported positive interactions with the agent, even hinting that it actually understood their psychological needs, as if it had the ability to represent their mental states. More recently, researchers examined the aptitude of large language models (LLMs) in completing classic tests of ToM reasoning and found that in at least some cases, they are achieving human-level capabilities (Strachan et al., 2024). Despite such impressive achievements in machine ToM, research is still needed to realize robust ToM for robots and other intelligent systems. For example, trivial alterations to classic ToM tasks can undermine the performance of LLM-based machine intelligences (Ullman, 2023). The goal of this Research Topic is two-fold: 1) improve the state-of-the-art ToM models adapted from cognitive science for robots and 2) advance new models of social cognition developed for the unique challenges of robots and intelligent systems. Both sub-goals are rich with research challenges.

## 2 Thematic areas

The papers in this Research Topic explore Theory of Mind from multiple perspectives, spanning human-robot coordination, assessment methodologies, and collective intelligence. The selected contributions demonstrate the breadth of contemporary ToM research, from engineering real-time collaborative systems to developing frameworks for benchmarking socio-cognitive abilities in artificial agents. These works examine ToM at multiple scales—from dyadic human-robot interactions to emergent dynamics in multi-agent teams—advancing both our theoretical understanding of mental state reasoning and its practical implementation in artificial intelligence and robotics.

### 2.1 Theory of mind for human-robot coordination

Effective human-robot collaboration requires agents to anticipate each other’s actions and achieve coordination with minimal explicit communication. The papers in this section explore computational mechanisms that enable artificial agents to reason about human mental states and leverage this understanding for seamless coordination. These contributions span sparse communication strategies, dynamic real-time coordination in heterogeneous teams, and flexible collaborative patterns that emerge without predefined roles.

Jiang et al. propose a relevance model grounded in decision theory and theory of mind to explain how humans select information for communication under real-time constraints (Jiang et al.). Tested in a simulated navigation task where participants and AI agents cooperatively avoid traps, the model accurately predicts human communication choices and outperforms the GPT-4 LLM in the same cooperative scenario. The work demonstrates that when humans receive assistance from an AI agent using the relevance model, they achieve significantly higher performance and provide higher ratings compared to a heuristic-based approach.

Nicolescu et al. expand simulation-based Theory of Mind approaches to enable collaborative task execution by heterogeneous teams of humans and multiple robots (Nicolescu et al.). Their distributed architecture addresses the challenge of dynamic coordination to avoid overlapping actions while ensuring correct task execution with hierarchical representations and multiple execution constraints. The system introduces a continuous-valued metric accounting for robots’ task execution capabilities during dynamic, online task allocation, validated through experiments with heterogeneous robot teams and human-robot teams performing household tasks under varying environmental conditions.

Schröder et al. introduce the concept of fluid collaboration (FC), characterized by frequent changes in task assumptions and resource consumption in response to varying environmental affordances (Schröder et al.). Through their Cooperative Cuisine (CoCu) environment inspired by a popular mobile game, they demonstrate that humans naturally engage in dynamically established collaboration patterns with minimal explicit communication, relying on efficient mentalizing. The authors argue for resource-rational and action-driven ToM reasoning integrated with action planning to enable artificial agents to effectively participate in fluid collaboration.

### 2.2 Assessing and benchmarking theory of mind in artificial systems

As ToM capabilities become increasingly central to artificial intelligence research, the field requires robust frameworks for evaluating and developing these capacities in computational systems. The papers in this section address this need by proposing novel assessment approaches and experimental platforms. One contribution investigates the computational modeling of higher-order ToM—moving beyond simple mental state attribution to reasoning about nested beliefs. The other presents a comprehensive developmental framework grounded in psychology that provides structured environments for studying socio-cognitive abilities in both reinforcement learning agents and LLMs.

Tavella et al. emphasize the current literature’s focus on first-order ToM models and investigate the potential for creating computational models of higher-order ToM. Higher-order ToM involves reasoning about nested mental states (e.g., “I think that you think that she believes …”), which is crucial for sophisticated social interactions throughout human development (Tavella et al.). By incorporating higher-order ToM in AI systems, artificial agents could better coordinate complex actions in domains such as warehouse logistics and healthcare, where understanding multiple layers of perspective-taking enhances collaborative performance.

Kovač et al. present The SocialAI School, a framework that leverages developmental psychology to study artificial socio-cultural agents (Kovač et al.). Drawing inspiration from Michael Tomasello and Jerome Bruner’s work on socio-cognitive development, they outline a broader set of concepts than typically studied in AI, including social cognition (joint attention, perspective taking), communication, social learning, formats, and scaffolding. Their tool offers a customizable suite of procedurally generated environments that can be used with both multimodal reinforcement learning agents and text-based LLMs, providing the AI community with a versatile platform for investigating how agents can enter, learn from, and contribute to a surrounding culture.

### 2.3 Theory of mind in team dynamics and collective intelligence

While individual ToM capabilities enable dyadic coordination, the complexity of multi-agent systems introduces emergent properties that arise from the interplay of multiple minds reasoning about each other. This section examines how ToM functions at the collective level, exploring how artificial social intelligence integrates into human team dynamics. This contribution bridges individual cognitive mechanisms with system-level outcomes, demonstrating that ToM’s impact extends beyond pairwise interactions to fundamentally shape team effectiveness and collective problem-solving.

Bendell et al. examine the integration of Artificial Social Intelligence (ASI) into human teams, focusing on how ASI can enhance teamwork processes in complex tasks (Bendell et al.). In their study, teams of three participants collaborated with ASI advisors designed to exhibit Artificial Theory of Mind (AToM) while engaged in an interdependent task. Using a profiling model to categorize teams based on taskwork and teamwork potential, they found that teams with higher potential in these dimensions had more positive perceptions of team processes and ASI advisors. Notably, while team performance mediated perceptions of team processes, perceptions of ASI advisors were positively correlated with team potential independent of performance outcomes, highlighting the need for ASI systems to be adaptable and responsive to specific team characteristics.

## 3 Future challenges

The papers in this Research Topic demonstrate significant progress in modeling, measuring, and applying ToM in artificial systems, but important challenges remain. For example, current AI approaches to ToM rely heavily on prompted or cue-based reasoning, where systems engage in mental state inference only when explicitly instructed to do so. As recent work has highlighted (Gurney et al., 2024), developing robust ASI will require moving beyond prompted ToM toward spontaneous ToM, i.e., reasoning about others’ mental states that emerges from unintentional cognitive processes rather than explicit task demands. This distinction is particularly crucial as machine intelligence fundamentally differs from human cognition in its learning mechanisms, experiential grounding, and the nature of its underlying computational processes, suggesting that achieving human-like social reasoning may require architectural innovations beyond scaling current generative models.

## 4 Conclusion

The contributions in this Research Topic illustrate the growing maturity of ToM research at the intersection of artificial intelligence, robotics, and cognitive science. From novel assessment methods to practical applications in human-agent coordination and insights into collective intelligence, these works demonstrate how ToM serves as a critical bridge between individual cognition and social interaction. As the field advances, continued interdisciplinary collaboration will be essential for developing artificial systems that not only reason about mental states when prompted, but engage in the kind of flexible, spontaneous social reasoning that characterizes human intelligence.

## Author disclaimer

The views and conclusions contained in this document are those of the authors and should not be interpreted as representing the official policies, either expressed or implied, of the Army Research Office or the U.S. Government. The U.S. Government is authorized to reproduce and distribute reprints for Government purposes notwithstanding any copyright notation herein.
